# Early Initiation of Antiretroviral Therapy Preserves the Metabolic Function of CD4^+^ T Cells in Subtype C Human Immunodeficiency Virus 1 Infection

**DOI:** 10.1093/infdis/jiad432

**Published:** 2023-10-06

**Authors:** Kewreshini K Naidoo, Andrew J Highton, Omolara O Baiyegunhi, Sindiswa P Bhengu, Krista L Dong, Madeleine J Bunders, Marcus Altfeld, Thumbi Ndung’u

**Affiliations:** HIV Pathogenesis Programme, The Doris Duke Medical Research Institute, University of KwaZulu-Natal, Durban, South Africa; Department of Virus Immunology, Leibniz Institute of Virology, Hamburg, Germany; Department of Microbiology and Immunology, University of Otago, Dunedin, New Zealand; Africa Health Research Institute, Durban, South Africa; HIV Pathogenesis Programme, The Doris Duke Medical Research Institute, University of KwaZulu-Natal, Durban, South Africa; Ragon Institute of Massachusetts General Hospital, Massachusetts Institute of Technology and Harvard University, Cambridge, Massachusetts, USA; Division of Infectious Diseases, Massachusetts General Hospital, Boston, Massachusetts, USA; Harvard Medical School, Boston, Massachusetts, USA; Department of Virus Immunology, Leibniz Institute of Virology, Hamburg, Germany; III Department of Medicine, University Medical Center Hamburg-Eppendorf, Hamburg, Germany; Department of Virus Immunology, Leibniz Institute of Virology, Hamburg, Germany; German Center for Infection Disease (DZIF), Partner Site Hamburg-Lübeck-Borstel-Riems, Germany; HIV Pathogenesis Programme, The Doris Duke Medical Research Institute, University of KwaZulu-Natal, Durban, South Africa; Africa Health Research Institute, Durban, South Africa; Ragon Institute of Massachusetts General Hospital, Massachusetts Institute of Technology and Harvard University, Cambridge, Massachusetts, USA; Division of Infection and Immunity, University College London, London, United Kingdom

**Keywords:** immune dysfunction, acute HIV-1 infection, CD4^+^ T cells, immunometabolism, antiretroviral therapy

## Abstract

**Background:**

Immune dysfunction often persists in people living with human immunodeficiency virus (HIV) who are on antiretroviral therapy (ART), clinically manifesting as HIV-1-associated comorbid conditions. Early ART initiation may reduce incidence of HIV-1–associated immune dysfunction and comorbid conditions. Immunometabolism is a critical determinant of functional immunity. We investigated the effect of HIV-1 infection and timing of ART initiation on CD4^+^ T cell metabolism and function.

**Methods:**

Longitudinal blood samples from people living with HIV who initiated ART during hyperacute HIV-1 infection (HHI; before peak viremia) or chronic HIV-1 infection (CHI) were assessed for the metabolic and immune functions of CD4^+^ T cells. Metabolite uptake and mitochondrial mass were measured using fluorescent analogues and MitoTracker Green accumulation, respectively, and were correlated with CD4^+^ T cell effector functions.

**Results:**

Initiation of ART during HHI prevented dysregulation of glucose uptake by CD4^+^ T cells, but glucose uptake was reduced before and after ART initiation in CHI. Glucose uptake positively correlated with interleukin-2 and tumor necrosis factor-α production by CD4^+^ T cells. CHI was associated with elevated mitochondrial mass in effector memory CD4^+^ T cells that persisted after ART and correlated with PD-1 expression.

**Conclusions:**

ART initiation in HHI largely prevented metabolic impairment of CD4^+^ T cells. ART initiation in CHI was associated with persistently dysregulated immunometabolism of CD4^+^ T cells, which was associated with impaired cellular functions and exhaustion.

## Teaser

This study reveals irregularities of cellular metabolism associated with immune dysfunction of CD4^+^ T cells in people living with human immunodeficiency virus, with and without antiretroviral therapy. However, antiretroviral therapy initiation in acute infection preserved most CD4^+^ T cell metabolic functions.

Human immunodeficiency virus (HIV)–associated immune activation results in progressive depletion and functional impairment of CD4^+^ T cells [1–3]. Antiretroviral therapy (ART) promotes quantitative and functional CD4^+^ T cell recovery in people living with HIV (PLWH), but reconstitution is often better in individuals who initiate ART early [4–6], although the specific mechanisms are not well defined. Cellular immune metabolism is integral to antiviral responses and there is evidence to suggest dysregulation of these functions during HIV-1 infection [7, 8]. Harnessing immunometabolism may therefore offer potential therapeutic strategies for ART-suppressed PLWH and necessitates better understanding of the relationship between cellular metabolism and immunity in the context of HIV-1 infection.

Quiescent naive and memory T cells rely on mitochondrial oxidative phosphorylation for cell maintenance and undergo substantial metabolic reprogramming to meet the energetic and biosynthetic demands of transitioning into effector T cells [7]. Findings of a few studies investigating cellular immune metabolism in PLWH suggest that CD4^+^ T cells gain a highly glycolytic profile characterized by increased expression of glucose transporter, hexokinase activity, and lactate production [9, 10], and exhibit impaired mitochondrial respiration that is associated with CD4^+^ T cell apoptosis and depletion [11–13]. Metabolic impairments of CD4^+^ T cells are only partially corrected by ART [10, 12, 13], and the impact of the timing of ART initiation on CD4^+^ T cell metabolism remains unknown. We investigated the consequences of ART initiation during hyperacute HIV-1 infection (HHI) or chronic HIV-1 infection (CHI) on the metabolic and immune function of CD4^+^ T cells in PLWH.

## METHODS AND MATERIALS

### Study Participants

Participants were from the Females Rising through Education, Support and Health (FRESH) [14, 15] or HIV Pathogenesis Programme Acute Infection cohorts [16]. Studies were approved by the Biomedical Research Ethics Committee of the University of KwaZulu-Natal. Participants gave written informed consent. Participants were grouped as HHI, initiating ART upon HIV-1 RNA detection (Fiebig stage I–II [HIV-1 RNA positive, p24 antigen negative/positive, and HIV-1 antibody negative]), or initiating ART during CHI (complete Western blot profile). Similar ART regimens were administered to treated HHI and CHI groups, with the exception of raltegravir, which was administered to individuals treated during HHI until 90 days after viral suppression (Supplementary Table 1). Cryopreserved peripheral blood mononuclear cell (PBMC) samples were obtained before and after ART. Controls were pre-infection PBMCs from the HHI group.

### Cellular Metabolite Analogue Uptake and Mitochondrial Mass Characterization

Metabolic assays were performed as described elsewhere [17–19]. PBMCs were cultured in glucose-free Roswell Park Memorial Institute 1640 medium supplemented with 50 μM 2-deoxy-2-(7-nitro-21,3-benzoxadiazol-4-yl)amino-D-glucose (2-NBDG [glucose]) or phosphate-buffered saline supplemented with 0.125 μM 4,4-difluoro-5,7-dimethyl-4-bora-3a,4a-diaza-s-indacene-3-hexadecanoic acid (BODIPY FL C_16_ [fatty acids]) for 30 minutes, or Hanks’ Balanced Salt Solution supplemented with 200 μM L-kynurenine (large neutral amino acids via SLC7A5) for 4 minutes at 37°C and 5% carbon dioxide (CO_2_). To assess mitochondrial mass (MM), PBMCs were incubated in R10-medium supplemented with 100 nM MitoTracker Green (MTG) for 30 minutes at 37°C and 5% CO_2_. Next, PBMCs were stained for viability and with CD3, CD4, CD8, CD14, CD19, CD45RA, and CCR7 antibodies, fixed with 1% paraformaldehyde, and acquired using a BD LSRFortessa cell analyzer. 2-NBDG, BODIPY FL C_16_ and MTG were detected in the fluorescein isothiocyanate (FITC) channel. L-kynurenine was detected in the Brilliant Violet (BV) 421 channel (Supplementary Table 2).

### Agilent Seahorse Cell Mito Stress Test

The Agilent Seahorse Cell Mito Stress Test (Agilent Technologies) was performed to measure the bioenergetic profiles of PBMC samples. Briefly, PBMCs were cultured in either R10-medium alone or supplemented with 1 μg each of purified anti-CD3 and anti-CD28 antibodies for 18 hours at 37°C and 5% CO_2_. Subsequently, PBMCs were resuspended in Cell Mito Stress Test medium, transferred to a Cell-Tak–coated Seahorse XFe96 cell culture microplate and incubated under CO_2_-free conditions at 37°C for 30 minutes. Extracellular flux was measured in a Seahorse Xfe96 analyzer in the presence of mitochondrial modulators, as described elsewhere [20]. Samples were measured in triplicate. Readings were normalized to the cell counts using the BioTek Cytation cell imager (Agilent) (Supplementary Table 2).

### Functional Intracellular Staining

PBMCs were cultured in R10-medium supplemented with 1 μg each of purified anti-CD3 and anti-CD28 antibodies and brefeldin A for 18 hours at 37°C and 5% CO_2_. PBMCs were subsequently stained for viability and with CD3, CD4, CD8, CD14, CD19, CD45RA, CCR7, HLA-DR, CD38, and PD-1 antibodies, fixed with FIX & PERM medium A, permeabilized with FIX & PERM medium B, and stained with interleukin-2 (IL-2) and tumor necrosis factor (TNF)-α antibodies, before acquisition using a BD LSRFortessa cell analyzer (Supplementary Table 2).

### Software and Statistical Analysis

FlowJo software, version 9.8.5, was used for flow cytometry data analysis. Seahorse Wave Desktop Software, version 2.6.0.31, was used for Seahorse Cell Mito Stress Test data analysis. GraphPad Prism software, version 9.0.1, was used for statistical analyses and graphical display. Kruskal-Wallis test with the Dunn's test for multiple comparisons was used for comparing >2 groups. Wilcoxon matched-pairs signed rank test and Mann-Whitney test were used for comparing 2 groups with paired and unpaired values, respectively. Associations were examined using Spearman rank correlation. Differences were considered statistically significant at *P* < .05.

## RESULTS

### Viral Suppression and CD4^+^ T Cell Recovery in Study Participants

To investigate the impact of ART timing on the metabolic function of T cells, participants initiating ART during HHI or CHI were assessed. Of these participants, 33 (91%) were female (100% in HHI and 83% in CHI groups). The median age at ART commencement (interquartile range [IQR]) was 21 (20–22) and 23 (20–24) years in the HHI and CHI groups, respectively (Table 1). In the HHI group, the median viral load (IQR) was 4.28 (3.89–5.31) log_10_ copies/mL at ART initiation, and this was suppressed below the detection limit within 2 months on ART (Supplementary Figure 1*A, left*). The median viral load (IQR) in the CHI group was 3.91 (3.61–4.24) log_10_ copies/mL before ART, with most participants suppressed within 3–4 months on ART (Supplementary Figure 1*A, right*).

**Table 1.** Participant Characteristics

		HHI				CHI		
Characteristic	HIV Uninfected	ART Naive	After 2 mo of ART	After 6 mo of ART	After 12 mo of ART	ART Naive	After 6 mo of ART	After 12 mo of ART
Sex	Female, n = 18					Female, n = 15; male, n = 3		
Age at ART initiation, years	21 (20–22)					23 (20–24)		
Viral load, log_10_ RNA copies/mL	NA	4.28 (3.89–5.31)	<20	<20	<20	3.91 (3.61–4.24)	<20	<20
CD4^+^ T cell count, cells/µL	927 (799–1020)	636 (448–800)	736 (656–986)	811 (618–917)	828 (732–1000)	599 (473–719)	677 (580–793)	752 (669–926)
CD8^+^ T cell count, cells/µL	551 (428–836)	431 (333–845)	477 (400–849)	470 (334–733)	585 (382–939)	841 (660–1135)	671 (512–778)	658 (530–769)

Data represent median (IQR) values. Abbreviations: ART, antiretroviral therapy; CHI, chronic HIV-1 infection; HHI, hyperacute HIV-1 infection; HIV, human immunodeficiency virus; IQR, interquartile range; NA, not applicable.

The median CD4^+^ T cell count (IQR) was 927 cells/µL (799–1020 cells/µL) in the HHI group before infection, declining to 636 cells/µL (448–800 cells/µL) within the first week of HIV-1 RNA detection (Supplementary Figure 1*B, left*), and 599 cells/µL (473–719 cells/µL) in the CHI group before ART (Supplementary Figure 1*B, right*), both significantly lower than in uninfected individuals (Supplementary Figure 1*C*). CD4^+^ T cell counts were comparable in uninfected and HIV-infected groups 12 months after ART; however, the dynamics of recovery differed. ART during HHI allowed for rapid recovery of CD4+ T cells within 2 months (Supplementary Figure 1*D, left*), whereas individuals with CHI showed gradual CD4^+^ T cell recovery after treatment, with recovery more rapid 6 months after ART initiation (Supplementary Figure 1*D, right*). Together, these data demonstrate rapid viral suppression when ART was initiated during HHI coupled with rapid CD4^+^ T cell recovery, while ART initiation during CHI required a longer time to fully suppress viremia and was associated with slower CD4^+^ T cell recovery.

### Compromised Metabolite Analogue Uptake by CD4^+^ T Cells in HIV-1 Infection with Partial Correction Following ART Initiation

**Figure 1.**
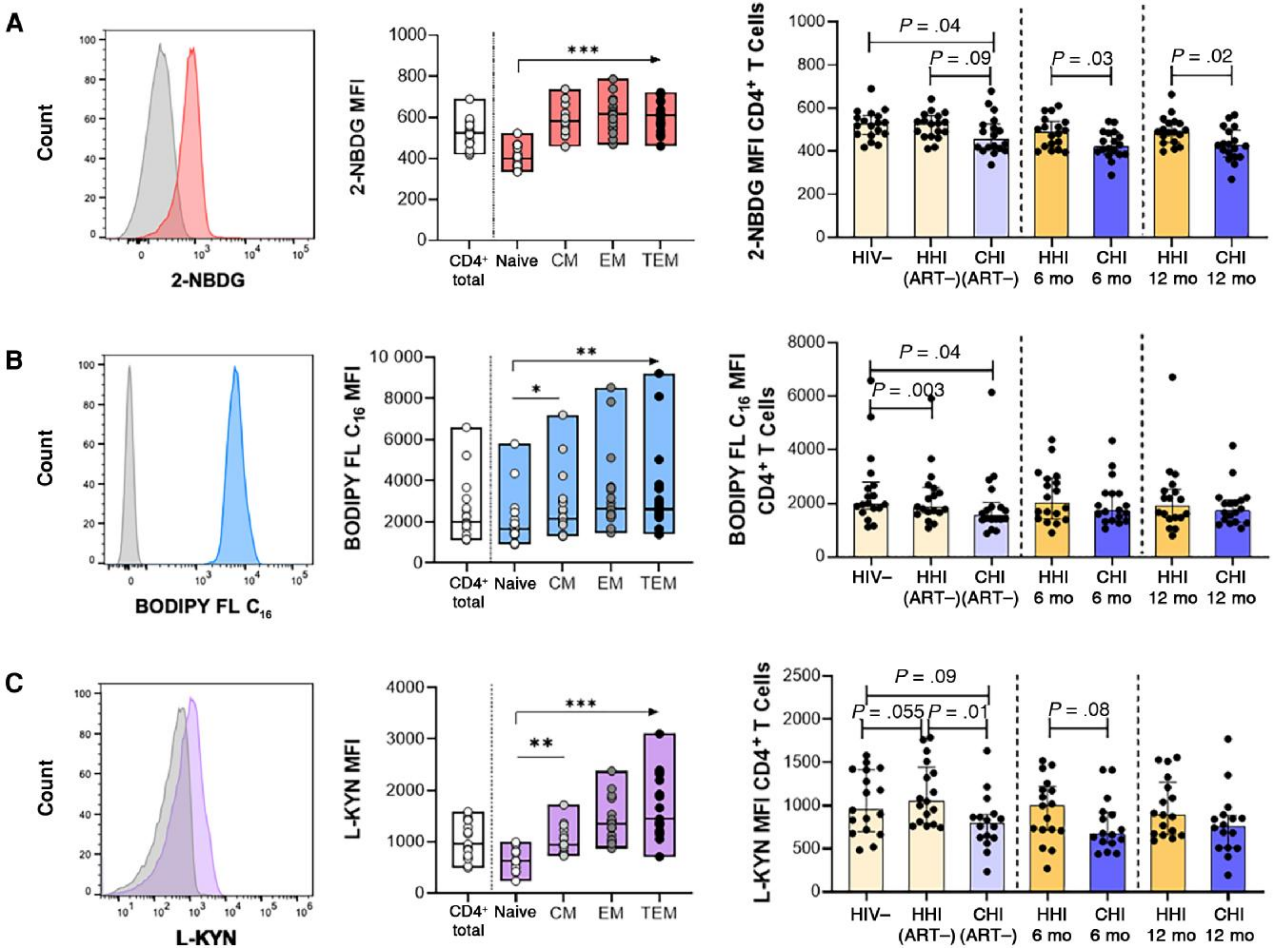
Compromised metabolite analogue uptake by CD4^+^ T cells in human immunodeficiency virus (HIV)-1 infection with partial correction following antiretroviral therapy (ART) initiation. *A, Left*, Representative histogram of 2-deoxy-2-(7-nitro-21,3-benzoxadiazol-4-yl)amino-D-glucose (2-NBDG) uptake (glucose) compared with Fluorescence Minus One (FMO) control in CD4^+^ T cells. *Middle,* 2-NBDG uptake in CD4^+^ T cells and subsets in the HIV-1–negative (HIV−) group. *Right,* Comparison of 2-NBDG uptake in CD4^+^ T cells between hyperacute HIV-1 infection (HHI) and chronic HIV-1 infection (CHI) groups before and after ART. *B*, *Left*, Representative histogram of 4,4-difluoro-5,7-dimethyl-4-bora-3a,4a-diaza-s-indacene-3-hexadecanoic acid (BODIPY FL C_16_) uptake (lipids) compared with FMO control in CD4^+^ T cells. *Middle,* BODIPY FL C_16_ uptake in CD4^+^ T cells and subsets in the HIV− group. *Right,* Comparison of BODIPY FL C_16_ uptake in CD4^+^ T cells between HHI and CHI groups before and after ART. *C*, *Left*, Representative histogram of L-kynurenine (L-KYN) uptake (amino acids) compared with FMO control in CD4^+^ T cells. *Middle,* L- KYN uptake in CD4^+^ T cells and subsets in the HIV− group. *Right,* Comparison of L-KYN uptake in CD4^+^ T cells between HHI and CHI groups before and after ART. Abbreviations: 6 mo, 6 months after ART initiation; 12 mo, 12 months after ART initiation; ART−, ART naive; CM, central memory; EM, effector memory; MFI, Median Fluorescence Intensity; TEM, terminal EM. *P < .05; **P < .01; ***P < .001.

Metabolic processes are integral to the optimal functioning of immune cells [7]. There is, however, a paucity of data on the consequences of HIV and the timing of ART initiation for immune cell metabolism. We therefore investigated T cell capacities to internalize essential nutrients in participant groups by flow cytometry (Supplementary Figure 2). Glucose, fatty acid, and amino acid uptake were measured using fluorescent metabolite analogues 2-NBDG, BODIPY FL C_16_, and L-kynurenine, respectively (Figure 1*A*–1*C, left*). In uninfected individuals, delineation by CD4^+^ T cell subsets showed that metabolite analogue uptake was relatively low in naive CD4^+^ T cells and that uptake was predominantly by memory subsets, particularly CD4^+^ effector memory T (T_EM_) and terminal effector memory T (T_TEM_) cells (Figure 1*A*–1*C, middle*).

Assessment was extended to HHI and CHI groups to determine the impact of HIV-1 infection and ART timing on metabolite analogue uptake by total CD4^+^ T cells (Figure 1*A*–1*C, right*). 2-NBDG uptake was comparable in individuals with HHI before and during early infection preceding ART and remained stable after ART (Figure 1*A, right*). In contrast, individuals with CHI displayed lower 2-NBDG uptake at baseline than uninfected individuals and those with HHI before ART, and this difference compared to the HHI group persisted up to 12 months on ART (Figure 1*A, right*). BODIPY FL C_16_ uptake by CD4^+^ T cells was significantly lower in both untreated HIV-1–infected groups than in uninfected individuals (Figure 1*B, right*), but improved after ART initiation so that BODIPY FL C_16_ uptake in HHI and CHI groups was similar to that in the HIV-1–negative group (*P* > .05 for all comparisons). L-kynurenine uptake by CD4^+^ T cells was largely unaffected by HIV-1 infection (Figure 1*C, right*). It should be noted, however, that although L-kynurenine uptake was not significantly affected by HIV infection, this does not exclude the possibility that uptake of other amino acids, such as glutamine, are affected owing to their uptake by other transporters [21].

**Figure 2.**
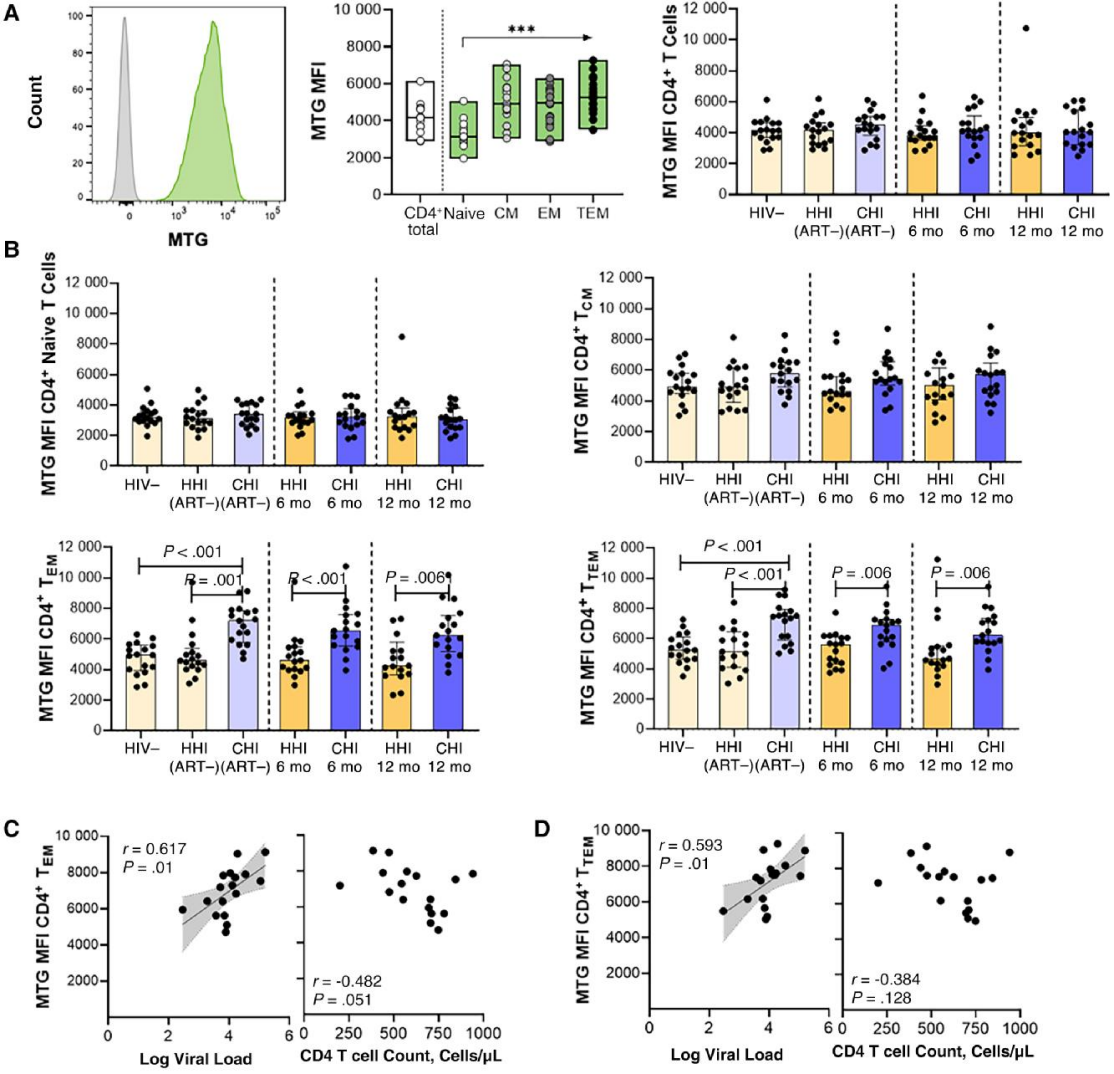
Higher mitochondrial mass in CD4^+^ effector memory T (EM) cell subsets in individuals starting antiretroviral therapy (ART) during chronic human immunodeficiency virus (HIV)-1 infection (CHI). *A*, *Left,* Representative histogram of MitoTracker Green FM dye (MTG) uptake compared with Fluorescence Minus One (FMO) control in CD4^+^ T cells. *Middle,* MTG uptake in CD4^+^ T cells and subsets in the HIV-1–negative (HIV−) group. *Right,* Comparison of MTG uptake in CD4^+^ T cells between groups with hyperacute HIV-1 infection (HHI) and CHI before and after ART. *B*, Comparison of MTG uptake in CD4^+^ T cell subsets, including naive (*top left*), central memory (CM; *top right*), EM (*bottom left*), and terminal EM (TEM; *bottom right*) CD4^+^ T cells, between HHI and CHI groups before and after ART. *C, D,* Correlations between MTG uptake in EM (*C*) and TEM (*D*) subsets in the CHI treated group before ART and viral load or CD4^+^ T cell count. ***P < .001. Abbreviations: 6 mo, 6 months after ART initiation; 12 mo, 12 months after ART initiation; ART−, ART naive; MFI, Median Fluorescence Intensity.

No associations were observed between total CD4^+^ T cell metabolite analogue uptake and viral load or CD4^+^ T cell count before ART initiation in HIV-infected groups (Supplementary Figure 3). Further analysis showed no significant differences in metabolite analogue uptake by total CD8^+^ T cells between PLWH before and after ART compared to uninfected individuals (Supplementary Figure 4*A*–4C). Lower 2-NBDG uptake in untreated CHI was observed in CD8^+^ T_EM_ cells (*P* = .004; data not shown); however, this function was reconstituted after ART initiation and was then comparable to 2-NBDG uptake by CD8^+^ T_EM_ cells in uninfected individuals (*P* = .13; data not shown). Collectively, the data showed only minor changes in CD8^+^ T cells but suggest that glucose and fatty acid uptake by CD4^+^ T cells is impaired during untreated HIV-1 infection, independent of viremia or CD4^+^ T cell count. Initiating ART during HHI preserved the uptake of both nutrients by CD4^+^ T cells. Starting ART during CHI improved fatty acid uptake to levels seen in uninfected individuals but did not fully rescue glucose uptake by CD4^+^ T cells, which remained significantly reduced even after 12 months of ART.

### Higher MM in CD4^+^ T_EM_ Cell Subsets in Individuals Starting ART During CHI

Most energy production occurs via oxidative phosphorylation within mitochondria. Optimal mitochondrial functioning is therefore key to effective energy production required for the regulation of T cell immune functions [22]. Mitochondrial dynamics within T cells during HIV-1 infection in relation to ART timing was determined using fluorescent MTG as a measure of relative MM and the quantity of active mitochondria (Figure 2*A, left*). In uninfected individuals, MM was higher in memory CD4^+^ T cells than in naive cells (Figure 2*A, middle*). In total CD4^+^ T cells, no significant differences in MM were detected during HIV-1 infection, before or after ART, compared to uninfected individuals (Figure 2*A, right*). CD4^+^ T cell subset analysis showed that HIV-1 infection and ART initiation did not significantly affect MM in naive and CD4^+^ central memory T (T_CM_) cells (Figure 2*B, top*). There was, however, substantially higher MM in both T_EM_ and T_TEM_ CD4^+^ T cell subsets in untreated individuals with CHI than in uninfected persons and those with HHI before ART (Figure 2*B, bottom*). Augmented MM of CD4^+^ T_EM_ and T_TEM_ T cells in treatment-naive individuals with CHI was strongly associated with viremia (Figure 2*C, left*, and 2*D, left*) and inversely correlated with CD4^+^ T cell counts (Figure 2*C, right*).

Although MM dropped after ART, it was still significantly higher in CHI treated individuals compared to HHI treated individuals after 12 months of ART (Figure 2*B, bottom*). Spearman analysis revealed that 2-NBDG uptake (glucose) positively correlated with MM in total CD4^+^ T cells in CHI treated individuals (*r* = 0.500; *P* = .04), but this association was not observed at the CD4^+^ T cell subset level (naive, *r* = 0.298 and *P* = .24; T_CM_, *r* = 0.339 and *P* = .18; T_EM_, *r* = 0.288 and *P* = .26; T_TEM_, *r* = 0.259 and *P* = .31; data not shown). Next, MTG signal was measured in CD8^+^ T cells; here, significantly higher MM was observed in untreated individuals with CHI, in total CD8^+^ T cells (Supplementary Figure 4*D*) and CD8^+^ T cell subsets (naive, *P* = .08; T_CM_, *P* = .01; T_EM_, *P* = .02; T_terminally differentiated effector memory cells re-expressing CD45RA_  _(TEMRA)_, *P* = .03; data not shown). Relative MM decreased after ART initiation in the CD8^+^ T cells of CHI treated individuals, and after 12 months of ART was comparable to that of uninfected individuals.

To further investigate mitochondrial changes induced by HIV infection, we performed the Cell Mito Stress Test to measure the oxygen consumption rate profiles in PBMCs from uninfected individuals and CHI treated individuals, either without or after T cell receptor (TCR) stimulation (Supplementary Figure 5*A*). Basal respiration tended to be higher in those with CHI before ART initiation for unstimulated and stimulated conditions (*P* = .055; data not shown), but no differences in maximal respiration were observed between uninfected and HIV-infected individuals (no stimulation, *P* = .17; TCR stimulated, *P* = .40; data not shown). Importantly, the percentage spare respiratory capacity, which reflects the reserve mitochondrial respiration capacity that is available to the cell under stress-induced conditions, was lower in individuals with CHI before and during ART, compared to the HIV-negative group (Supplementary Figure 5*B*), suggestive of persistent mitochondrial dysregulation in individuals that initiated ART during CHI. Together, the data show reduced mitochondrial spare respiratory capacity of PBMCs and elevated MM in CD4^+^ T_EM_ and T_TEM_ cells associated with viremia in CHI that persisted after ART-induced viral suppression, indicating dysregulated mitochondrial activity in CHI treated individuals, even after 12 months on ART.

### Association Between Metabolic Dysregulations and Repressed Immune Functions of CD4^+^ T Cells in Individuals Initiating ART During CHI

**Figure 3.**
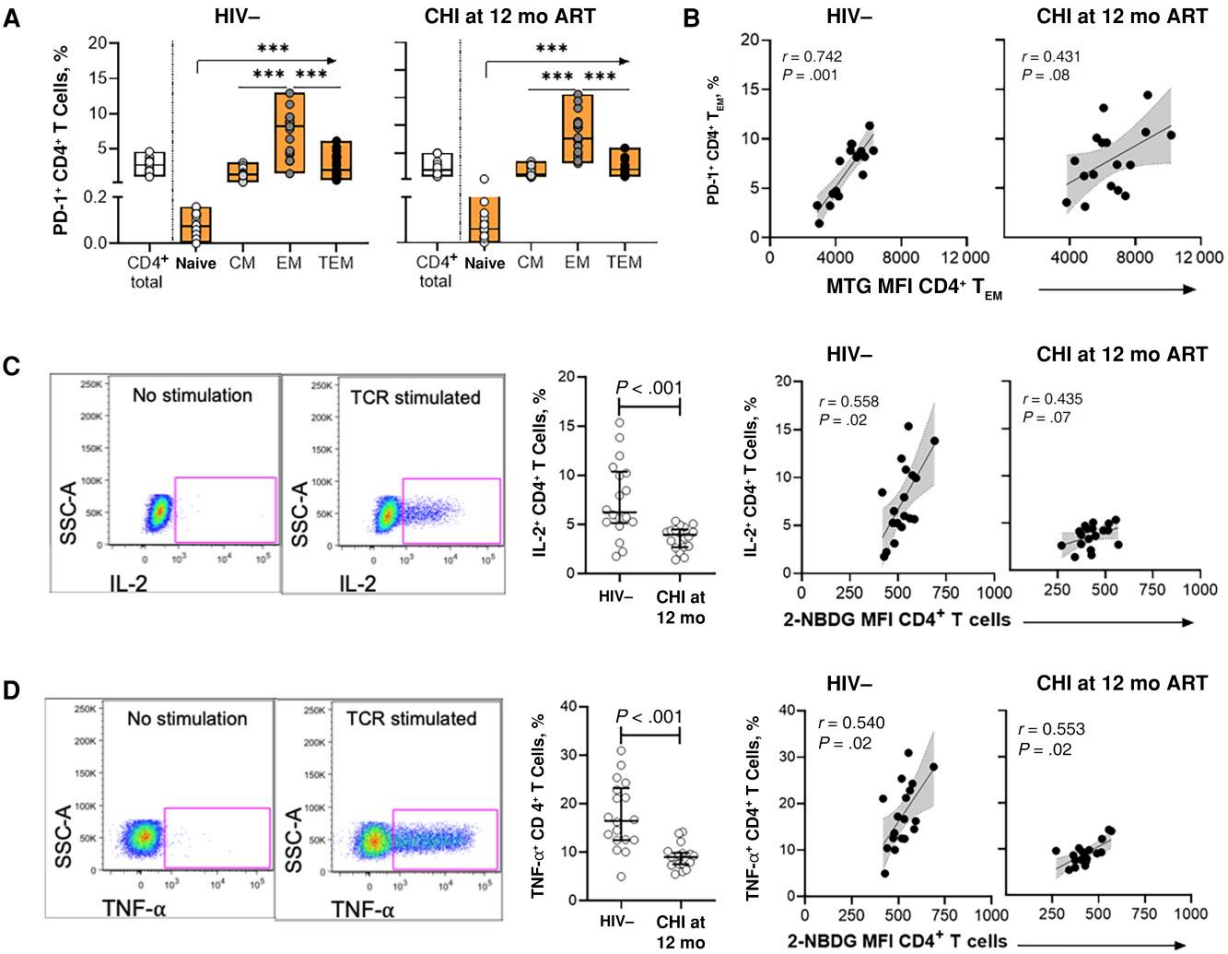
Metabolic dysregulations are associated with reduced cytokine production and cellular exhaustion of CD4^+^ T cells in individuals initiating antiretroviral therapy (ART) during chronic human immunodeficiency virus (HIV)-1 infection (CHI). *A*, Frequency of PD-1^+^ CD4^+^ T cells and subsets in the HIV-1–negative (HIV−) group (*left*) and CHI treated group at 12 months after ART initiation (*right*). *B*, Correlations between frequency of PD-1^+^ CD4^+^ effector memory T (T_EM_) cells and MitoTracker Green FM dye (MTG) uptake in CD4^+^ T_EM_ cells. *C*, *Left,* Representative gating strategy showing interleukin-2 (IL-2) production by CD4^+^ T cells without or with stimulation. *Middle,* Comparison of IL-2 production by T cell receptor (TCR)–stimulated CD4^+^ T cells between the HIV− group and the CHI treated group at 12 months after ART initiation. *Right,* Correlations between IL-2 production and glucose uptake by CD4^+^ T cells in the HIV− group and the CHI treated group at 12 months after ART initiation. *D*, *Left,* Representative gating strategy showing tumor necrosis factor (TNF)-α production by CD4^+^ T cells without or with stimulation. *Middle,* Comparison of TNF-α production by TCR-stimulated CD4^+^ T cells between the HIV− group and the CHI treated group after 12 months of ART. *Right,* Correlations between TNF-α production and glucose uptake by CD4^+^ T cells in the HIV− group and the CHI treated group after 12 months of ART. ***P < .001. Abbreviations: 12 mo, 12 mo after ART initiation; CM, central memory; MFI, Median fluorescence intensity; SSC, side scatter; TEM, terminal EM.

After observing metabolic dysregulations of CD4^+^ T cells in CHI that persisted up to 12 months after ART initiation, we assessed the impact of altered glucose uptake and MM on CD4^+^ T cell immune functions. CD4^+^ T cells were assessed for coinhibitory receptor PD-1 expression that is essential for lymphocyte homeostasis signaling and is associated with T cell exhaustion in HIV-1 infection [23, 24]. PD-1 expression was highest in memory CD4^+^ T cells, particularly CD4^+^ T_EM_ cells, in uninfected individuals (Figure 3*A, left*) and CHI individuals after 12 months of ART (Figure 3*A, right*). Furthermore, PD-1 expression in CD4^+^ T_EM_ cells was significantly higher in CHI compared to uninfected controls (*P* = .007; data not shown).

We therefore focused subsequent analysis on potential associations between PD-1 expression and metabolic dynamics of CD4^+^ T_EM_ cells in uninfected individuals and CHI treated individuals in whom we had observed persistent metabolic dysregulations. The percentage of PD-1^+^ CD4^+^ T_EM_ cells strongly correlated with MM (*r* = 0.612; *P* < .001) yet only weakly associated with 2-NBDG uptake (*r* = 0.211; *P* = .12; data not shown) of CD4^+^ T_EM_ cells. Further assessment of MM and PD-1-expression in CD4^+^ T_EM_ cells in the different groups revealed a positive correlation in uninfected individuals but also in individuals with CHI before and after treatment, in which both MM and the percentage of PD-1^+^ CD4^+^ T_EM_ cells were higher (Figure 3*B*). These data demonstrate that elevated MM in CD4^+^ T_EM_ cells observed in CHI was associated with up-regulation of the exhaustion marker PD-1.

We next assessed the functional ability of CD4^+^ T cells to respond to TCR stimulation and correlated these effector functions with glucose uptake and MM. PBMCs cultured with purified anti-CD3 and anti-CD28 antibodies were measured for IL-2 and TNF-α production by CD4^+^ T cells using flow cytometry (Figure 3*C, left*, and 3*D, left*). Lower percentages of CD4^+^ T cells produced IL-2 (Figure 3*C, middle*]) and TNF-α (Figure 3*D, middle*) in individuals with CHI compared to uninfected individuals, even after 12 months of ART. Furthermore, the percentages of both IL-2– and TNF-α–producing CD4^+^ T cells positively correlated with 2-NBDG uptake by CD4^+^ T cells in the HIV-1–negative group and the CHI group 12 months after ART initiation (Figure 3*C, right*, and 3*D, right*), in line with the need to generate energy through glycolysis for cytokine production. Collectively, the data show that glucose uptake and associated cytokine production were impaired in individuals starting ART during CHI, even after 12 months of ART. In contrast, MM of CD4^+^ T cells were not correlated with the percentage of cytokine-producing CD4^+^ T cells (Supplementary Figure 6), indicating distinct but synergistic consequences of persistent impairments of glucose uptake and MM in CD4^+^ T cells on function and expression of checkpoint inhibitors.

## DISCUSSION

We explored the consequences of HIV-1 infection and ART timing on the metabolic function of T cells in PLWH from South Africa. The timing of ART initiation was associated with distinct viral load and CD4^+^ T cell recovery dynamics, with rapid virus suppression and faster CD4^+^ T cell rebound when ART was started in HHI. However, after 12 months of ART, CD4^+^ T cell counts in both HIV-treated groups were comparable to those of uninfected individuals, suggestive of quantitative CD4^+^ T cell reconstitution irrespective of ART timing. Despite this, we provide evidence that metabolic functioning of CD4^+^ T cells during HIV-1 infection is highly dependent on the timing of ART. Using metabolite surrogates, we demonstrate that ART initiation during HHI preserved nutrient uptake and MM of CD4^+^ T cells. In contrast, untreated CHI was associated with compromised nutrient uptake by CD4^+^ T cells and higher MM in CD4^+^ T_EM_ cells. Starting ART during CHI improved lipid uptake by CD4^+^ T cells to levels observed in uninfected individuals, but reduced glucose uptake and elevated MM in CD4^+^ T cells persisted following ART. Assessment of these metabolic dysregulations in relation to immune function revealed that impaired 2-NBDG uptake was associated with lower capacity of CD4^+^ T cells to produce effector cytokines, while higher MM correlated with up-regulated PD-1 expression in CD4^+^ T_EM_ cells in CHI treated individuals. These results demonstrate that the timing of ART initiation can affect the metabolic reconstitution of CD4^+^ T cells.

Metabolism of essential nutrients is central to immune cellular functioning and dependent on nutrient availability [25, 26]. We report impediment of 2-NBDG and BODIPY FL C_16_ uptake by CD4^+^ T cells, suggestive of impaired glucose and fatty acid uptake, in untreated CHI. Limited studies exploring the metabolic capacities of CD4^+^ T cells from PLWH have focused on markers of glucose metabolism [9, 12]. Intensified glycolysis in CD4^+^ T cells has been demonstrated by an up-regulation of glucose transporter and higher 2-NBDG internalization during treatment-naive CHI, and was not fully remedied by ART [9, 10]. Comparing the metabolic findings reported here with past studies necessitates caution. Sex and age disparities of cohorts that can affect metabolism regulation, immunity, and antiviral responses need to be considered, with predominantly young female participants with relatively high CD4^+^ T cell counts included in the current study, compared to previous studies that included mainly older all-male cohorts, often with lower CD4^+^ T cell counts [27–29]. Furthermore, the metabolic dysregulations resulting from different ART regimens in PLWH need to be considered (reviewed in [30]). Similar ART regimens were administered to HHI and CHI treated groups, with the exception of raltegravir, which was administered to HHI treated individuals until 90 days after viral suppression; however, raltegravir has not previously been associated with severe metabolic perturbations after short-term therapy [31], and we did not observe any persistent metabolic dysregulations in individuals that started treatment during HHI. Other differences, including ethnicity and diet, may also influence differential metabolic programming during infection [32], and the lack of clinical information regarding body mass index, blood glucose levels, and diabetes diagnoses in study participants represents a limitation of the current study. Importantly, our study design enabled us to assess the impact of ART timing on nutrient uptake of CD4^+^ T cells. ART initiation during HHI prevented impairments of nutrient uptake in CD4^+^ T cells, while starting ART during CHI only enabled reconstitution of fatty acid internalization while impaired glucose uptake by CD4^+^ T cells persisted.

Glucose is a critical nutrient for all stages of cellular respiration. Reduced glucose uptake by CD4^+^ T cells when ART was started during CHI may therefore affect mitochondria, the powerhouses of cellular respiration. MM was highest within CD4^+^ T cell memory subsets versus naive cells irrespective of HIV status. No differences were observed in the MM of total CD4^+^ T cells between CHI treated PLWH and uninfected counterparts, consistent with previous findings [10]; however, significantly higher accumulation of mitochondria in the CD4^+^ T_EM_ cells of treatment-naive persons with CHI was observed and was positively correlated with viremia, suggestive of disrupted homeostatic mitochondrial biogenesis in response to chronic antigenic stimulation. There was only incremental waning of MM following ART, such that elevated MM in these cellular subsets of CHI treated people was still observed 12 months after ART initiation, suggesting that once viral replication is suppressed, the mitochondrial activity of CD4^+^ T_EM_ cells in CHI treated individuals takes longer to stabilize. Mitochondrial respiration levels in CD4^+^ T cells were previously found to be lower before and after ART-controlled infection [12, 13], providing insight into the detrimental effects of HIV-1 on mitochondrial dynamics in CD4^+^ T cells that are not resolved with ART. Our data show that early ART initiation has an advantageous impact in preserving the mitochondrial dynamics of CD4^+^ T cells, an observation that might explain the described clinical benefit of early ART initiation [33].

Indications of compromised glucose uptake and MM modulation in CD4^+^ T cells of chronically treated PLWH led us to investigate the effect of these metabolic irregularities on CD4^+^ T cell functionality. TCR-induced cytokine production, specifically for IL-2 and TNF-α, was significantly inferior in CHI treated individuals, even after 12 months on ART. Impaired cytokine production significantly associated with reduced CD4^+^ T cell glucose uptake, in line with glucose being a key metabolite for effector molecule production [34]. Furthermore, PD-1 expression was up-regulated on CD4^+^ T_EM_ cells in all groups, and higher MM was associated with higher frequencies of PD-1–expressing CD4^+^ T_EM_ cells in CHI treated individuals before and after ART. Antigen persistence induces the up-regulation of immune checkpoint inhibitors, including PD-1, in T cells in an effort to attenuate persistent signaling downstream of the TCR. In chronic lymphocytic choriomeningitis virus mouse models, PD-1 signaling during early T cell exhaustion exerted glycolytic and mitochondrial alterations that ultimately dysregulated mitochondrial biogenesis [35]. Thus, dysregulated MM in CD4^+^ T_EM_ cells in PLWH due to chronic immune activation and higher PD-1 signaling may represent an early precursor marker to cellular exhaustion.

Collectively our data reveal metabolic mechanisms that account for variable functional recovery of CD4^+^ T cells in a population group with high HIV-1 infection burden in sub-Saharan Africa; and highlight the beneficial effects of early ART administration in averting metabolic alterations to CD4^+^ T cells, at least within the first year of ART. The study emphasizes the complexity of immunometabolism that warrants further investigation and may require population-specific approaches for complementary therapeutic interventions in ART-suppressed PLWH.

## Supplementary Data

Supplementary materials are available at *The Journal of Infectious Diseases* online. Consisting of data provided by the authors to benefit the reader, the posted materials are not copyedited and are the sole responsibility of the authors, so questions or comments should be addressed to the corresponding author.

## Supplementary Material

jiad432_Supplementary_Data

## Notes

***Author contributions.*** Concept and study design: K. L. D., M. J. B., M. A., and T. N. Data collection, experimental optimization and acquisition: K. K. N., A. J. H., O. O. B., S. P. B., and K. L. D. Analysis and interpretation of data: K. K. N., M. A., and T. N. Drafting of the manuscript: K. K. N., M. A., and T. N.

***Financial support.*** This study was supported by the Deutsche Forschungsgemeinschaft (grant BU 3630/2–1 to M. J. B., M. A., and T. N.), the Bill & Melinda Gates Foundation (grants OPP1212883 and INV-033558 to T. N.), Gilead Sciences Inc. (grant 00406 to K. L. D. and T. N.), and the International AIDS Vaccine Initiative (grant UKZNRSA1001 to T. N.). This research was also funded in part by Wellcome [Grant number Wellcome Strategic Core award: 201433/Z/16/A]. For the purpose of open access, the authors have applied a CC BY public copyright licence to any Author Accepted Manuscript version arising from this submission.
