# Supplementary material for: Early Initiation of Antiretroviral Therapy Preserves the Metabolic Function of CD4+ T Cells in Subtype C Human Immunodeficiency Virus 1 Infection
Source: J Infect Dis. 2023 Oct 6;229(3):753–62. doi: 10.1093/infdis/jiad432 (PMC10938216; doi:10.1093/infdis/jiad432)
Supplement: jiad432_Supplementary_Data [file jiad432_supplementary_data.docx]

**Early initiation of antiretroviral therapy preserves the metabolic function of CD4+ T-cells in subtype C HIV-1 infection**

Kewreshini K. Naidoo^1,2^, Andrew J. Highton^3^, Omolara O. Baiyegunhi^4^, Sindiswa P. Bhengu^1^, Krista L. Dong^5,6,7^, Madeleine J. Bunders^2,8^, Marcus Altfeld^2,9^, Thumbi Ndung’u^1,4,5,10*^

^1^HIV Pathogenesis Programme, The Doris Duke Medical Research Institute, University of KwaZulu-Natal, South Africa.

^2^Department of Virus Immunology, Leibniz Institute of Virology, Germany.

^3^Department of Microbiology and Immunology, University of Otago, New Zealand.

^4^Africa Health Research Institute, Durban, South Africa.

^5^Ragon Institute of Massachusetts General Hospital, Massachusetts Institute of Technology and Harvard University, Cambridge, MA, USA.

^6^Division of Infectious Diseases, Massachusetts General Hospital, Boston, MA, USA.

^7^Harvard Medical School, Boston, MA, USA.

^8^III Department of Medicine, University Medical Center Hamburg-Eppendorf, Germany.

^9^German Center for Infection Disease (DZIF), Partner Site Hamburg-Lübeck-Borstel-Riems, Germany.

^10^Division of Infection and Immunity, University College London, United Kingdom.

***Correspondence:** Thumbi Ndung’u (thumbi.ndungu@ahri.org)

Africa Health Research Institute, K-RITH Tower Building, Level 3, Nelson R. Mandela School of Medicine, 719 Umbilo Road, Congella, Durban, 4001, South Africa.

| **Supplementary Table 1** | | |
| --- | --- | --- |
| **Clinical test** | **Assay / Kit** | **Supplier** |
| CD4+ T-cell count | BD Trucount, analysed on a four-parameter FACSCalibur flow cytometer | Becton Dickinson |
| Viral load | NucliSENS EasyQ HIV-1 v2.0 kit | BioMérieux |
| HIV-1 p24 antigen / antibodies to HIV-1 | Elecsys HIV combi PT 4th Generation (Ag+Ab test) p24 Ag | Roche |
| Western Blot | GS HIV-1 Western blot kit | Bio-Rad Laboratories |
| **Participant group** | **ARV regimen** | |
| Hyperacute HIV Infection (HHI) | FDC pill (300 mg tenofovir disoproxil fumarate, 200 mg emtricitabine, 600 mg efavirenz) and raltegravir (400 mg twice a day until 90 days after viral suppression i.e. <20 copies per ml) (n=18) | |
| Chronic HIV  Infection (CHI) | Atenef (n=1), Atroiza (n=13), Odimune (n=3) or Tribuss (n=1) (all 300 mg tenofovir disoproxil fumarate, 200 mg emtricitabine, 600 mg efavirenz) | |

| **Supplementary Table 2** | | | | |
| --- | --- | --- | --- | --- |
| **Reagent** | | | **Supplier** | **Cat Number** |
| 2-deoxy-2-[(7-nitro-2,1,3-benzoxadiazol-4-yl)amino]-D-glucose (2NBDG) | | | Cayman Chemical | 11046 |
| 4,4-Difluoro-5,7-Dimethyl-4-Bora-3a,4a-Diaza-s-Indacene-3-Hexadecanoic Acid (BODIPY FL C_16_) | | | Invitrogen | D3821 |
| L-kynurenine | | | Sigma-Aldrich | K8625 |
| MitoTracker Green FM (MTG) | | | Invitrogen | M7514 |
| RPMI 1640 Medium, no glucose | | | Gibco | 11879020 |
| R10-medium (RPMI 1640 Medium supplemented with 10% FBS, 1% Penicillin-Streptomycin, 1% L-Glutamine, 1% HEPES buffer) | | | Prepared in-house | n/a |
| RPMI 1640 Medium | | | Gibco | 11875119 |
| Fetal Bovine Serum (FBS) | | | Gibco | F0679 |
| L-Glutamine | | | Thermo Scientific | 25030024 |
| Penicillin-Streptomycin (10,000 U/mL) | | | Gibco | 1514012 |
| HEPES (1 M) | | | Gibco | 15630080 |
| Trypan Blue | | | Bio-Rad Laboratories | 1450013 |
| Dulbecco’s Phosphate Buffered Saline (DPBS) | | | Gibco | 14190250 |
| Hanks′ Balanced Salt solution (HBSS) | | | Sigma-Aldrich | H6648 |
| Brefeldin A | | | Sigma-Aldrich | B6542 |
| LIVE/DEAD™ Fixable Aqua Dead Cell Stain Kit, for 405 nm excitation | | | Invitrogen | L34957 |
| LIVE/DEAD™ Fixable Near-IR Dead Cell Stain Kit, for 633 or 635 nm excitation | | | Invitrogen | L10119 |
| FIX & PERM™ Cell Permeabilization Kit | | | Invitrogen | GAS003 |
| BD CompBeads Anti-Mouse Ig, κ/Negative Control Compensation Particles Set | | | BD Biosciences | 552843 |
| Sphero Rainbow Fluorescent Particles, 3.0-3.4 µm (mid-range FL1 fluorescence) | | | BD Biosciences | 556298 |
| DPBS, no calcium, no magnesium | | | Gibco | 14190094 |
| Sodium bicarbonate (NaHCO₃) | | | Sigma | S5761 |
| Corning® Cell-Tak^TM^ Cell and Tissue Adhesive | | | Corning | CLS354240 |
| Dulbecco′s Modified Eagle′s Medium (DMEM) | | | Sigma | D5030 |
| Sodium chloride (NaCl) | | | Sigma | S9888 |
| HEPES solution | | | Sigma | H0887 |
| GlutaMAX™ Supplement | | | Gibco | 35050061 |
| Sodium pyruvate solution | | | Sigma | S8636 |
| D-(+)-Glucose | | | Sigma | G8270 |
| Phenol Red | | | Sigma | P3532 |
| CMST medium ( DMEM, 30mM NaCl, 5mM HEPES, 2mM GlutaMAX and 1mM sodium pyruvate, 10mM D-(+)-Glucose, 42µM phenol red; pH 7.4) | | | Prepared in-house | n/a |
| Oligomycin from *Streptomyces diastatochromogenes* | | | Sigma | O4876 |
| Carbonyl cyanide 4-(trifluoromethoxy)phenylhydrazone (FCCP) | | | Sigma | C2920 |
| Antimycin A from *Streptomyces* sp. | | | Sigma | A8674 |
| Rotenone | | | Sigma | 557368 |
| Hoechst 33342 Solution | | | Thermo Scientific | 62249 |
| Formaldehyde solution | | | Sigma | 252549 |
| Seahorse XFe96 FluxPak | | | Agilent Technologies | 102416-100 |
| **Antibody** | **Conjugate** | **Clone** | **Supplier** | **Cat Number** |
| anti-human CD3 | purified | OKT3 | BioLegend | 317304 |
| anti-human CD28 | purified | CD28.2 | BioLegend | 302933 |
| anti-human CD3 | BV 650 | OKT3 | BioLegend | 317324 |
| anti-human CD4 | APC | SK3 | BioLegend | 344614 |
| anti-human CD4 | PE/Cy5 | RPA-T4 | BioLegend | 300510 |
| anti-human CD8 | FITC | RPA-T8 | BD Biosciences | 555366 |
| anti-human CD8 | PE/Cy7 | SK1 | BD Biosciences | 335822 |
| anti-human CD14 | APC/Cy7 | HCD14 | BioLegend | 325620 |
| anti-human CD14 | V500 | M5E5 | BD Biosciences | 561391 |
| anti-human CD19 | APC/Cy7 | H1B19 | BioLegend | 302218 |
| anti-human CD19 | BV 510 | H1B19 | BioLegend | 302242 |
| anti-human CD45RA | APC/Cy7 | HI100 | BioLegend | 304128 |
| anti-human CD45RA | BV 711 | HI100 | BioLegend | 304138 |
| anti-human CD197 (CCR7) | PE/Dazzle 594 | G043H7 | BioLegend | 353236 |
| anti-human CD38 | PE | HB-7 | BioLegend | 356604 |
| anti-human HLA-DR | BV 711 | L234 | BioLegend | 307644 |
| anti-human CD279 (PD-1) | BV 421 | EH12.2H7 | BioLegend | 329920 |
| anti-human IL-2 | FITC | 5344.111 | BD Biosciences | 340448 |
| anti-human TNF-⍺ | PerCP/Cy5.5 | Mab11 | BioLegend | 502926 |
| **Software** | | | **Company** | |
| FlowJo version 9.8.5 | | | BD Life Sciences | |
| GraphPad Prism version 9.0.1 | | | GraphPad Software, LLC | |
| Seahorse Wave Desktop Software | | | Agilent Technologies | |

**
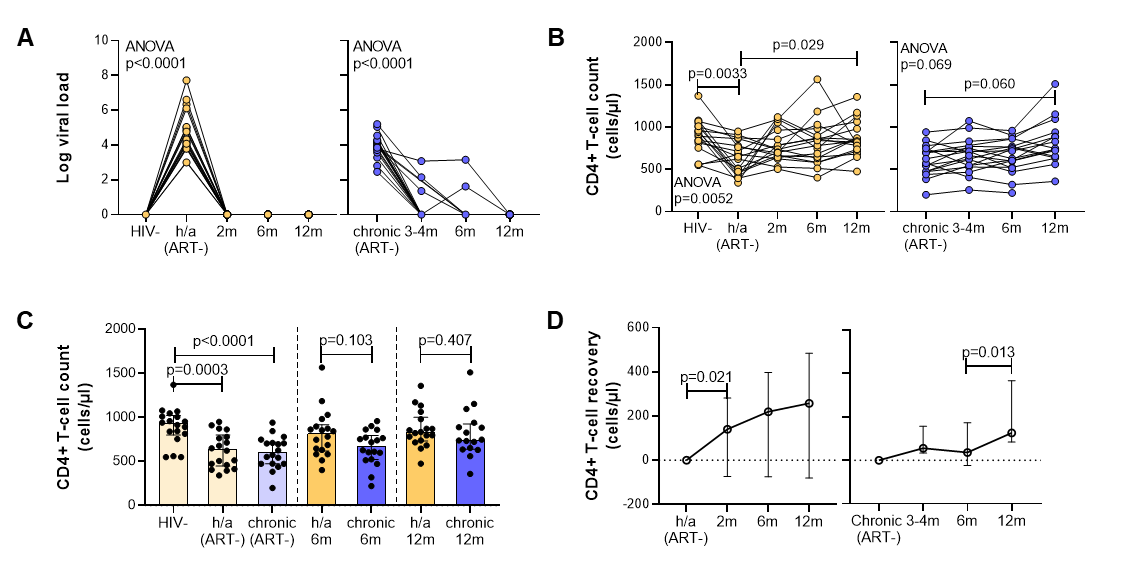
**

**Supplementary Figure 1: Viral suppression and CD4+ T-cell recovery in participant groups.**

(A) Log viral load and (B) CD4+ T-cell count pre- and post-treatment in groups initiating ART during hyperacute [left] or chronic [right] HIV-1 infection. (C) Comparison of CD4+ T-cell count between groups pre- and post-ART. (D) CD4+ T-cell recovery in groups initiating ART during hyperacute [left] and chronic [right] HIV-1 infection. Abbreviations: hyperacute [h/a]; ART naïve [ART-]; 2-months post-ART [2m]; 3-4-months post-ART [3-4m]; 6-months post-ART [6m]; 12-months post-ART [12m].

**Supplementary Figure 2: Representative gating strategy to identify total CD4+T-cells and CD4+T-cell subsets.** Side scatter area (SSC-A) vs forward scatter area (FSC-A) plots used to identify lymphocytes and exclude doublets by forward scatter height (FSC-H) vs FSC-A. Lymphocytes were stained for viability, anti-CD14 and anti-CD19 to exclude non-viable cells, monocytes and B cells, respectively. Anti-CD3 staining was used for T-cell gating followed by identification of CD4+T-cells and CD4+T-cell subsets (naïve, central memory [CM], effector memory [EM] and terminal effector memory [TEM]) using anti-CD45RA and anti-CCR7.


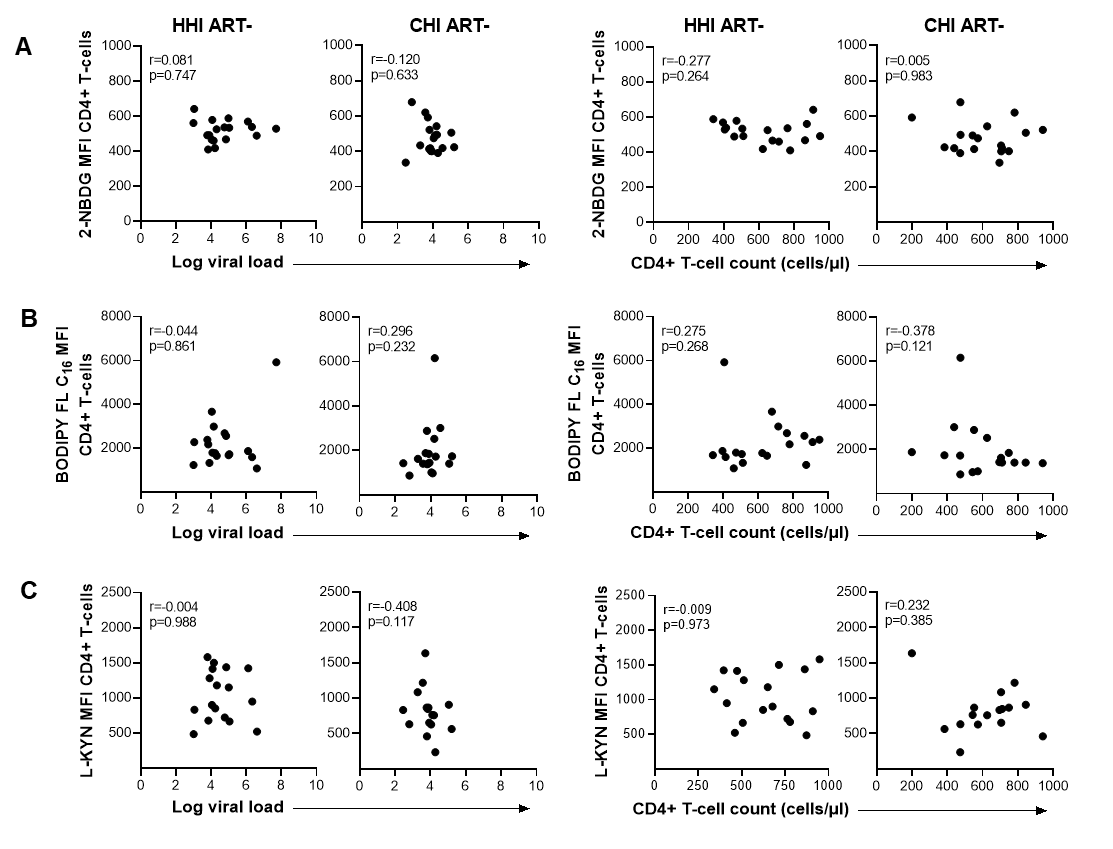


**Supplementary Figure 3: Correlations between metabolite analogue uptake and log viral load or CD4+ T-cell count.** (A) 2-NBDG, (B) BODIPY FL C_16_, and (C) L-KYN correlated to log viral load [left panel] and CD4+ T-cell count [right panel] in HHI and CHI individuals prior to ART initiation. Abbreviations: hyperacute HIV infection [HHI]; chronic HIV infection [CHI]; ART naïve [ART-]; L-kynurenine [L-KYN].

**** ****

**Supplementary Figure 4: Metabolite analogue uptake and mitochondrial mass of CD8+ T-cells.** (A) 2-NBDG, (B) BODIPY FL C_16_, (C) L-KYN and (D) MTG uptake by CD8+ T-cells in study groups. Abbreviations: L-kynurenine [L-KYN]; MitoTracker Green FM dye [MTG]; hyperacute [h/acute]; ART naïve [ART-]; 6-months post-ART [6m]; 12-months post-ART [12m].


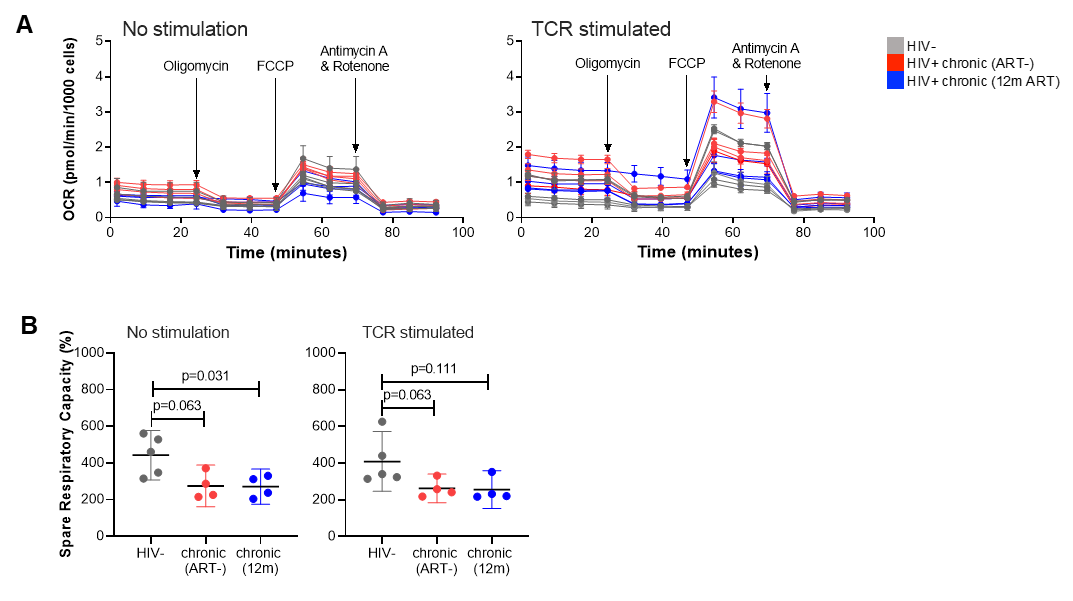


**Supplementary Figure 5: Bioenergetic profiles of PBMC using the** **Cell Mito Stress Test** (A) Oxygen consumption rate profiles in HIV negative individuals and CHI-treated individuals before and on 12-months ART. (B) Comparison of mitochondrial SRC between HIV-1 negative individuals and CHI-treated individuals before and on 12-months ART. Abbreviations: ART naïve [ART-]; 12-months post-ART [12m]; T-cell receptor [TCR]; Spare Respiratory Capacity [SRC].


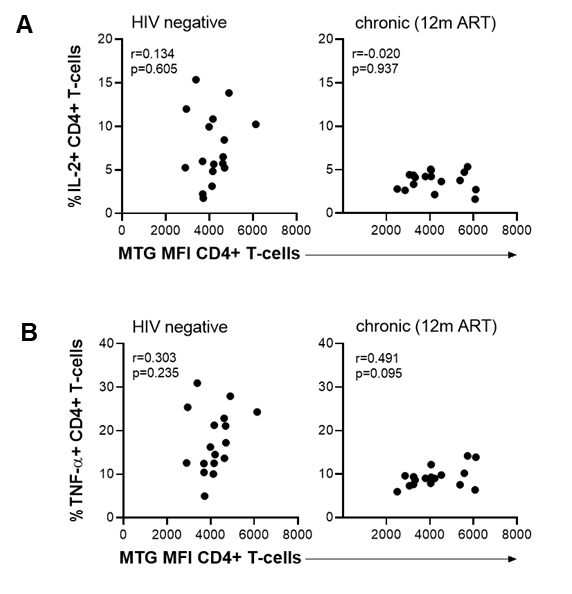


**Supplementary Figure 6: Correlations between cytokine production and mitochondrial mass of CD4+ T-cells.** (A) Correlations between IL-2 production and MTG uptake of CD4+ T-cells in the HIV-1 negative group [left] and the chronic treated group at 12-months post-ART [right]. (B) Correlations between TNF-⍺ production and MTG uptake of CD4+ T-cells in the HIV-1 negative group [left] and the chronic treated group at 12-months post-ART [right]. Abbreviation: MitoTracker Green FM dye [MTG].
